# Case Report: *RNF213* variant and choroidal anastomosis as potential risk factors for early stroke in moyamoya syndrome associated with Down syndrome

**DOI:** 10.3389/fped.2023.1289554

**Published:** 2023-11-02

**Authors:** Keisuke Yamamoto, Yasuyuki Kaku, Hiroshi Koga

**Affiliations:** ^1^ Department of Pediatrics, National Hospital Organization Beppu Medical Center, Beppu, Japan; ^2^ Department of Neurosurgery, Graduate School of Medical Sciences, Kumamoto University, Kumamoto, Japan

**Keywords:** cerebrovascular accident, collateral circulation, moyamoya disease, trisomy 21, ubiquitin-protein ligases

## Abstract

**Introduction:**

Recent studies have suggested associations between *RNF213* variants and the formation of periventricular anastomosis among patients with moyamoya disease, leading to early onset of cerebral hemorrhage and rebleeding.

**Case description:**

We report herein the case of a boy with Down syndrome and moyamoya syndrome. Exome sequencing identified a heterozygous *RNF213* R4810K variant. After ischemic stroke occurred at 9 years old, indirect surgical revascularization was performed for the left cerebral hemisphere and improved ischemic symptoms and cerebral hypoperfusion, while the left choroidal anastomosis remained. At 13 years old, he presented with left thalamic hemorrhage attributed to the anterior choroidal artery, with rebleeding observed four days after the initial hemorrhage under strict blood pressure control. The patient was discharged without neurological deficits 20 days after the hemorrhagic stroke.

**Conclusion:**

Presence of an *RNF213* variant and choroidal anastomosis may represent risk factors for cerebral hemorrhage in patients with Down syndrome and moyamoya syndrome, as well as in patients with moyamoya disease.

## 1. Introduction

Moyamoya disease (MMD) is recognized as a cerebrovascular disorder leading to ischemic and hemorrhagic stroke and occurs more commonly in East-Asian countries, with annual incidences of 0.5–1.5 per 100,000 individuals, compared to 0.1 per 100,000 in other regions (1). This is partly explained by a high frequency of the *RNF213* R4810K variant, the major susceptibility gene for MMD, among both patients with MMD (approximately 80%) and the general population (approximately 2%) in Japan and South Korea (1). Most pediatric patients with MMD are susceptible to ischemic attack, and the incidence of hemorrhagic attack increases with age (2). Antiplatelet therapy and/or surgical revascularization are commonly used to prevent future stroke (1). In addition, a recent Japanese randomized controlled trial of adult patients with hemorrhagic MMD showed that direct revascularization after initial hemorrhage had preventive effects against rebleeding, with an annual decrease of 4.9% per year (3).

Down syndrome, the most common chromosomal trisomy and recently called Down syndrome regression disorder (4), is well known to be associated with coexisting moyamoya arteriopathy or moyamoya syndrome (1). Moyamoya syndrome is a cerebrovascular disorder similar to MMD, but diagnostically distinguishable from MMD (5). Patients with Down syndrome and moyamoya syndrome (DS-MMS) are more likely to present with ischemic stroke (15.3%) and less likely to present with hemorrhagic stroke (2.7%) than patients with MMD alone (11.5% and 6.8%, respectively) (6). Blood pressure elevation has been reported as a foreshadowing symptom in patients with DS-MMS (7). The association between the development of cerebral collateral circulation and subsequent hemorrhagic attack among patients with DS-MMS remains to be elucidated because of limited data, with a recent systematic review including only four cases of DS-MMS with hemorrhagic stroke (8). In addition, the effectiveness of surgical treatment after hemorrhagic stroke has not been reported among DS-MMS patients.

We report herein the case of a boy with DS-MMS who developed hemorrhagic stroke after indirect revascularization and discuss factors contributing to hemorrhagic stroke in early adolescence among patients with DS-MMS.

## 2. Case description

**Figure 1:**
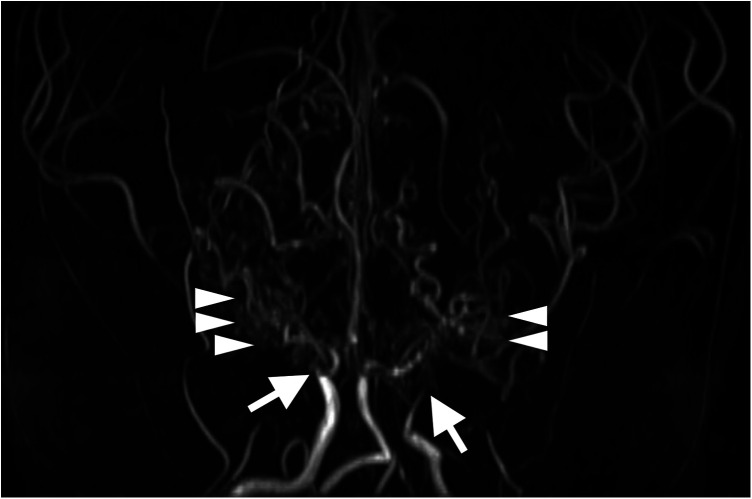
Three-dimensional time-of-flight magnetic resonance angiography at 8 years old shows bilateral occlusion of the supraclinoid internal carotid arteries (arrows) and development of collateral arteries (arrowheads).

**Figure 2:**
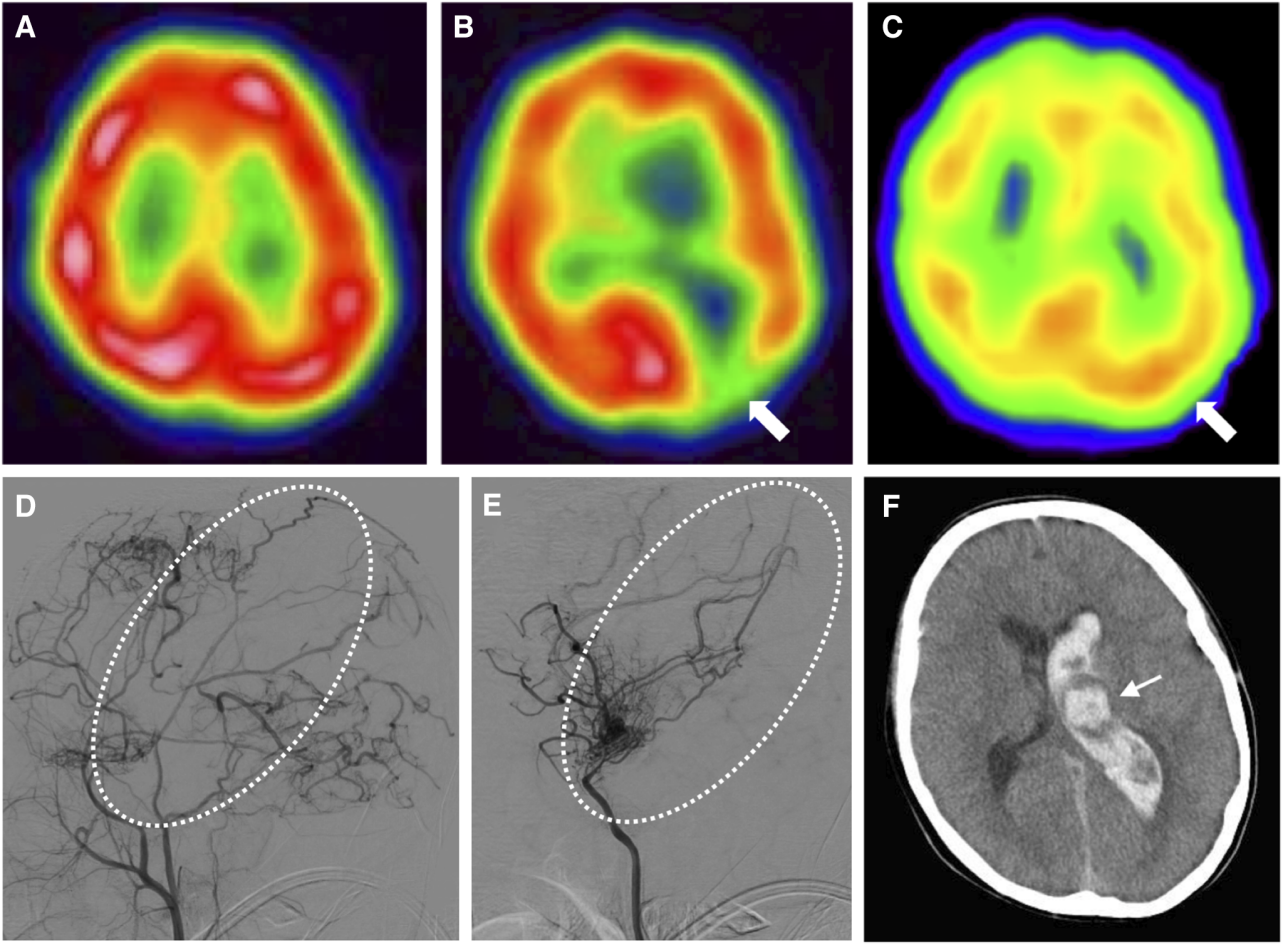
Time series of radiological features in this case. (**A**) N-isopropyl-p-[^123^I]iodoamphetamine single-photon emission computed tomography at 8 years old shows no cortical hypoperfusion. (**B**) Single-photon emission computed tomography at 9 years old shows hypoperfusion in the left occipital lobe (arrow). (**C**) Single-photon emission computed tomography at 11 years old (2 years after indirect revascularization) shows improved perfusion (arrow). (**D**) Left external carotid angiography at 12 years old shows sufficient blood supply to the left hemisphere via transdural anastomosis, but insufficient blood supply to the left central region (dotted circle). (**E**) Left internal carotid angiography at 12 years old shows the left central region with blood flow supplied by choroidal anastomosis (dotted circle). (**F**) Computed tomography of the brain at 13 years old shows left thalamic (arrow) and intraventricular hemorrhage, attributed to the dilated left anterior choroidal artery.

An 8-year-old Japanese boy presented with recurrent headache. He had a medical history of hypothyroidism and Down syndrome confirmed by a 47,XY, +21 karyotype. No thyroid peroxidase or anti-thyroglobulin antibody were detected (9). There was no family history of cerebrovascular disease. Magnetic resonance imaging and angiography showed old cerebral infarction in the left temporal lobe and bilateral occlusion of the terminal portions of the internal carotid arteries with development of collateral arteries consistent with moyamoya syndrome (Figure 1). Based on the asymptomatic stage and the lack of perfusion defects as revealed by ^123^I-iodoamphetamine single-photon emission computed tomography (SPECT) (Figure 2A), we initiated antiplatelet therapy with aspirin, not revascularization.

At 9 years old, he developed altered consciousness and slurred speech. Hypoperfusion was identified in the left cerebral hemisphere on ^123^I-iodoamphetamine SPECT (Figure 2B). To prevent further stroke, the patient underwent left-sided indirect revascularization consisting of encephalo-duro-arterio-synangiosis and encephalo-myo-synangiosis. No perioperative complications were observed. After the bypass surgery, ischemic symptoms regressed. Postoperatively, ^123^I-iodoamphetamine SPECT and cerebral angiography demonstrated improved cortical perfusion (Figures 2C, D). However, cerebral angiography showed left choroidal anastomosis and residual moyamoya vessels (Figure 2E).

**Figure 3:**
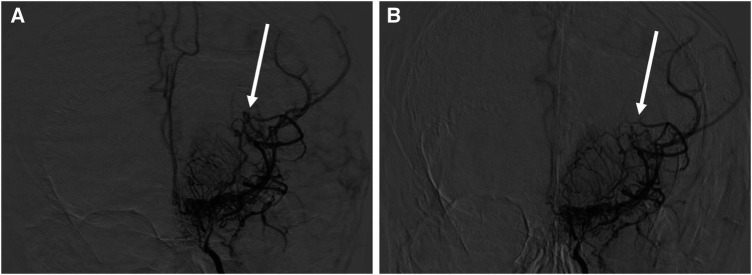
Angiographic features before and after hemorrhagic stroke in this case. (**A**) Left internal carotid angiography at 12 years old before hemorrhagic stroke shows dilation of the left anterior choroidal artery (arrow). (**B**) Left internal carotid angiography 4 months after hemorrhagic stroke shows spontaneous regression of the dilated left anterior choroidal artery (arrow).

At 13 years old, the patient was admitted to our center with sudden onset of severe headache and recurrent vomiting, with a Glasgow Coma Scale of 14 and blood pressure of 127/81 mmHg. Computed tomography (CT) of the brain revealed left thalamic hemorrhage with left intraventricular hemorrhage (Figure 2F), considered as a cerebral hemorrhage attributable to the left dilated anterior choroidal artery (10). Aspirin therapy was discontinued and blood pressure was managed using an intravenous calcium channel blocker for 7 days to achieve a systolic blood pressure under 120 mmHg (<95th percentile for age and sex) (11). Four days after the onset of cerebral hemorrhage, he again complained of headache. Brain CT at this point revealed expansion of intracerebral hemorrhage to the right ventricle, suggesting rebleeding. Eight days after onset, clinical symptoms improved and magnetic resonance imaging demonstrated decreased intracerebral hemorrhage. The patient developed bacterial aspiration pneumonia and gastric ulcer and was therefore started on intravenous ampicillin and famotidine. Twenty days after onset, brain CT detected no findings of post-hemorrhagic hydrocephalus and he was discharged with no neurological deficits. Four months later, cerebral angiography showed spontaneous regression of the dilated left anterior choroidal artery (Figures 3A, B).

Genomic DNA was extracted from the peripheral blood sample of the patient and targeted Sanger sequencing confirmed a heterozygous *RNF213* R4810K variant (GenBank accession number, NM_001256071.3), as a major susceptibility gene for MMD (12). Follow-up angiography detected the development of a contralateral right choroidal anastomosis and the patient therefore underwent right-sided direct revascularization to prevent future hemorrhagic stroke six months after onset of the left hemorrhagic stroke. Four months after revascularization, angiography showed regression of the right choroidal collateral vessels. No ischemic or hemorrhagic stroke was observed as of the time of writing, 22 months postoperatively.

## 3. Discussion

This study reports the case of a boy with DS-MMS who carried the *RNF213* R4810K variant and developed cerebral hemorrhage at 13 years old. Despite prior indirect revascularization, a choroidal anastomosis had formed and resulted in hemorrhagic stroke. Considering the epidemiological data, our patient developed hemorrhagic stroke at a younger age than expected (2). Presence of the *RNF213* variant and the development of choroidal anastomosis might have contributed to the early onset of ischemic and hemorrhagic stroke.

Choroidal anastomosis, as one of the major forms of periventricular collateral circulation in MMD, is commonly observed in patients with hemorrhagic MMD (10). Abnormal collateral vessels associated with cerebral hemorrhage were classified into three types: lenticulostriate anastomosis, thalamic anastomosis, and choroidal anastomosis (10). Choroidal anastomosis connects the choroidal arteries to medullary arteries and perfuses the cerebral cortex via retrograde flow through the medullary arteries. The Japan Adult Moyamoya (JAM) Trial revealed an association between cerebral hemorrhage and fragile choroidal anastomosis based on angiography, resulting in choroidal anastomosis being considered a risk factor for posterior hemorrhage (10). This finding is consistent with the clinical characteristics observed in our patient. The JAM Trial also reported that MMD patients with cerebral hemorrhage attributable to perforating arteries from the choroidal artery or posterior cerebral artery were at higher risk of rebleeding (annual incidence, 17.1%) than those from the anterior or middle cerebral artery (annual incidence, 3.0%) (13). Direct revascularization within 12 months after hemorrhagic attack has been shown to be beneficial in reducing the risk of rebleeding (3). In our patient, no additional left-sided direct revascularization was performed after the cerebral hemorrhage because of the spontaneous regression of the left choroidal anastomosis as a potential risk of rebleeding. After the development of a right choroidal anastomosis was confirmed, our patient underwent right-sided revascularization based on reports of collateral vessel regression following revascularization (14). Regression was indeed observed in our patient following revascularization.

Our patient with DS-MMS developed hemorrhagic stroke at a young age of 13 years, although intracranial hemorrhage usually occurs after 25 years old in patients with MMD (1, 2). In addition, a large epidemiological study comprising 518 DS-MMS patients in the United States demonstrated that hemorrhagic stroke was markedly less common (0.16%) than ischemic stroke (18.0%; *P *< 0.05) when restricted to patients <18 years old (6). Angiographic assessment in 37 patients with MMD and hemorrhagic stroke revealed dilation of the anterior choroidal artery in four of five (80%) patients <20 years old and in 23 of 32 (72%) ≥20 years old (15). Given these epidemiological data, the choroidal anastomosis found in our patient may have predisposed the patient to early-age hemorrhagic stroke.

Blood pressure elevation has been described in patients with MMD and DS-MMS, presumably as a systemic compensatory response against cerebrovascular stenosis (7). In addition, blood pressure reduction in patients with acute intracerebral hemorrhage reportedly correlates with attenuation of cerebral hematoma (16). Elevated blood pressure may be a driving factor for the development of hemorrhagic stroke in patients with MMD and DS-MMS.

The concept of *RNF213*-related vasculopathy has been proposed as a spectrum of intracranial artery disease (1). The *RNF213* gene encodes ring finger protein 213, also called mysterin, which has E3 ubiquitin ligase activity (17). The *RNF213* R4810K variant found in our patient likely contributed to early onset of ischemic stroke. A meta-analysis of genotype-phenotype correlation studies among East-Asian patients with MMD and the *RNF213* R4810K variant showed that early onset before 15 years old was more commonly observed in those homozygous (21/26, 81%; *P *= 0.003) or heterozygous (288/713, 40%; *P *= 0.001) for the variant than in those without the variant (326/1502, 22%) (12). A genetic analysis of 260 Chinese MMD patients elucidated an association between heterozygosity for the *RNF213* R4810K variant and the formation of choroidal anastomosis and other periventricular anastomoses (18). Another Chinese study reported an association between the *RNF213* R4810K variant and lenticulostriate anastomosis (19). The *RNF213* R4810K variant is thus associated with early onset of ischemic stroke through the formation of abnormal cerebral anastomoses and may have played a similar role in the pathogenesis of DS-MMS in our patient. Among a European population, early onset of MMD at ≤3 years old and extracerebral occlusive vasculopathy have been reported in patients with other genetic variants of *RNF213* (17). However, the role of *RNF213* dysfunction in the pathogenesis of MMD has not yet been fully elucidated.

Thus, recent studies have suggested associations between *RNF213* variants and the formation of periventricular anastomosis, including choroidal anastomosis, leading to early onset of ischemic or hemorrhagic symptoms and between choroidal anastomosis and cerebral hemorrhage and rebleeding among MMD patients (3, 10, 12, 13, 15, 17, 18). The present report indicates that these risk factors for the development of stroke found in patients with MMD alone may also be relevant in DS-MMS. *RNF213*-related vasculopathy is potentially more implicated in the pathogenesis of MMD and moyamoya syndrome than expected. Although both DS-MMS and *RNF213*-related vasculopathy are well-known disease entities, reports on potential associations between the two remain limited (20, 21). Further studies are needed to investigate the potentially synergistic effects of trisomy 21 and *RNF213* variants on the development of DS-MMS, to provide insights into the pathogenesis of moyamoya syndrome.

## Data Availability

The original contributions presented in the study are included in the article and further inquiries can be directed to the corresponding authors.
